# A universal state and its relaxation mechanisms of long-range interacting polygons

**DOI:** 10.1038/s41467-019-09795-6

**Published:** 2019-04-15

**Authors:** Hongchuan Shen, Hua Tong, Peng Tan, Lei Xu

**Affiliations:** 1 Department of Physics and State Key Laboratory of Surface Physics, Fudan University, 200433 Shanghai, China; 2 Department of Physics, The Chinese University of Hong Kong, Hong Kong, China; 3 Institute of Industrial Science, University of Tokyo, 4-6-1 Komaba, Meguro-ku Tokyo, 153-8505 Japan

## Abstract

Using polygonal magnetic particles, we conduct experiments to explore the space-filling properties of anisotropic blocks with long-range interactions. In contrast to previous studies, we obtain the surprising finding that our systems’ structures do not depend on the shape of building blocks: a single state, the hexagonal plastic crystal, appears as a universal attractor for a wide range of different polygons. This robust particle-shape independency appears as the interactions go beyond nearest neighbors. Particle shape plays an essential role in system relaxation, and determines the basic relaxation dynamics through a microscopic control parameter, internal roughness, produced by particle vertices. Thus our study reveals a new pattern-forming paradigm, in which particle shape plays little role in the static structure but determines the essential relaxation dynamics. Due to the ubiquity of long-range interactions and anisotropic building blocks, our discovery may shed new light on diverse problems involving structure formation, self-assembly, and packing.

## Abstract (web summary)

Space packing is not only of scientific interests, but also relevant to our daily life. Here, Shen et al. show that the interaction beyond the nearest neighbors leads to the formation of a universal structure, namely hexagonal plastic crystal, which is insensitive to the shape of building blocks.

## Introduction

How to fill up space with identical building blocks? Across different research fields, this classical question attracts the attention of mathematicians, physicists, chemists, and materials scientists. At first glance, it may seem to be a simple issue of fitting the local feature of individual blocks into the global space. However, in practice this issue is the basis for realizing desired solid structures in condensed matter physics, achieving novel self-assembly structures in materials science, and even understanding the formation of complex living colonies and assemblies in biological research^1–3^.

Among many control parameters, the shape of building blocks is of fundamental importance: it can influence the packing efficiency as demonstrated by the famous experiment of packing ellipsoidal M&M’s candies versus its spherical counterparts^4^, it may produce unique frustration structures as illustrated by depositing special ‘kite’ tiles under gravity^5^, and it could even help to realize highly-unusual phases such as quasicrystals with specially-designed blocks^6^.

Another important factor is the interactions among particles: by coupling fine-tuned interactions with carefully-designed particle shapes, different building blocks may assemble into various types of structures including liquid and plastic crystals^7–9^, superlattices^10–12^, quasi-crystals^6,13,14^, and glass^5^. Besides static structures, the coupling between shape and interaction also strongly influences dynamic responses^15–17^, and determines the system relaxation properties such as plasticity, friction, lubrication, and melting behaviors^18–27^.

Despite wide variations, most previous studies however share one common feature: the interactions are short-ranged and the building blocks can only influence their nearest neighbors. As a result, the entropic effect due to geometric constraints from neighboring blocks plays an essential role in structure formation^28–30^, which has led to amazing systems such as directional bonding and chiral structures^31–35^, open lattices^36,37^, and clathrate structures^38^. However, the opposite regime of coupling long-range interactions with anisotropic building blocks remains largely unexplored (note that ‘long-range interactions’ in this work mean the interactions going beyond the nearest neighbors). In this open regime dominated by non-entropic effect, are there any new physics, novel structures and unconventional material properties? This fundamental question remains to be addressed from both experimental and theoretical fronts.

In general, numerous studies have demonstrated that when the building blocks change their shapes, the system structures will vary correspondingly, with no universal state for different shapes^39–42^. Taking regular polygons as the simplest anisotropic model system, it is natural to expect different packing structures corresponding to different polygon shapes, for example the square lattice corresponding to square blocks and the triatic phases^43^ corresponding to triangular particles. System structure depending on particle shape seems to be a common sense in the field.

Strikingly, however, in this work we demonstrate that such a ‘common sense’ is actually due to the short-range interactions only between nearest neighbors. Once the interactions become long range and more neighbors get involved, we uncover a universal state—the hexagonal plastic crystal—for various polygonal systems. Across a broad density range, this state exists for hexagons, pentagons, squares and triangles, demonstrating robust shape independency in the long-range interaction regime. Although making little impact on the static structure, the particle shape however strongly influences the dynamic relaxations: under external perturbations different polygons relax distinctively, relying predominantly on the ‘internal roughness’ produced by polygon vertices (i.e., particle shapes). In particular, a novel relaxation mechanism via defect loop, which typically appears in quasicrystals^44^, surprisingly occurs in our crystal structure of triangles. Thus our study reveals a fundamentally new paradigm, in which particle shape plays little role in the static structure but determines the essential relaxation dynamics. Due to the ubiquity of long-range interactions and anisotropic building blocks, our discovery may shed new light on diverse problems involving structure formation, self-assembly, and packing^45,46^.

## Results

### Experimental design

One major reason for the poor understanding in the long-range and anisotropic interaction regime is the lack of a good experimental platform for single-particle-level measurements: molecules are typically anisotropic but too small to track individually, and colloids are large enough to visualize but difficult to achieve long-range anisotropic interactions^47–49^. To tackle this issue, we construct a two-dimensional (2D) system composed by hundreds to thousands of millimeter-sized magnetic polygon particles. This 2D system can be perturbed by a magnetic plate underneath, which moves back and forth parallel to the system plane, and a stable state can be achieved after tens to hundreds of such perturbations (see ‘Experimental Details’ in Methods). The magnetic repulsion among polygon particles can extend beyond multiple particle size and realize the long-range anisotropic interaction (see Supplementary Fig. 2). Because the interaction is long range, the number density of a ‘condensed state’, in which particles can ‘feel’ each other, could vary by 20 times. Such a large variation in density has rarely been achieved in previous short-range interacting systems. We also emphasize that particles can never contact each other directly throughout our density range, to avoid the strong contact interaction dominating the soft long-range repulsion, which would then resemble the short-range interaction regime extensively studied before (See Supplementary Fig. 4 right column for the highest density situations).

### A universal state formed by particle centers

**Fig. 1:**
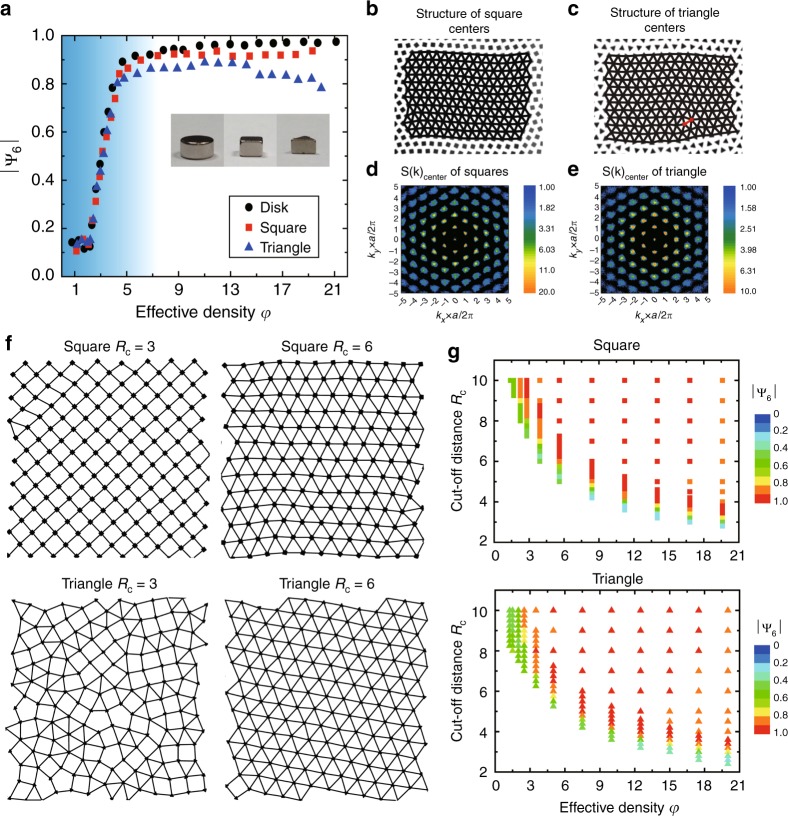
A universal state in different polygonal systems. **a** The global bond orientational order parameter |Ψ_6_| calculated from all particle centers versus particle density *φ* for high-symmetry (discs), intermediate symmetry (squares) and low-symmetry (triangles) particles. For all three groups, the system is disordered at low densities but ordered at high densities. **b** and **c** a universal hexagonal state is formed by particle centers in both square and triangle systems. We connect all particle centers to give a better illustration. The effective area densities are *φ* = 16.8 and *φ* = 14.2, respectively. **d**, **e** Fourier transform of particle centers in **c** and **d**. The length scale *a* is the average particle distance. **f** numerical simulations of squares (*φ* = 17.6) and triangles (*φ* = 15) illustrate that the states are shape dependent at a low cut-off distance but shape-independent at a high cut-off distance. Upper panels: square particles produce a square lattice at low cut-off distance *R*_*C* _= 3 (the unit of *R*_*C*_ is the disc particle’s diameter, i.e., 6 mm); while a hexagonal state appears at high cut-off distance *R*_*C* _= 6. Lower panels: triangle particles exhibit a glassy state at *R*_*C* _= 3 but a hexagonal state at *R*_*C* _= 6. **g** square and triangle particle’s phase diagrams from simulations. At large enough *R*_*C*_, a hexagonal state universally appears. The lower-left blank area is the region at and below the jamming point, where the system starts to unjam and fall apart

To obtain a systematic understanding on the effect of particle shape, we gradually break up the particle’s rotational symmetry: starting from discs, the particles are systematically varied into hexagons, pentagons, squares and triangles, as shown in Fig. 1a inset. Behaviors can be categorized into three typical groups: the high-symmetry group (discs and hexagons), the intermediate-symmetry group (pentagons and squares), and the low-symmetry group (triangles). For each group, we pick one representative particle shape and demonstrate the system’s overall order at different effective density *φ*, in Fig. 1a. The effective density *φ* is defined in such a way that at about *φ* = 1, the mutual repulsion just overcomes the friction and particles start to ‘feel’ each other (more experimental details are shown in Methods). The system’s overall order is quantified by the global six-fold bond orientational order parameter |Ψ_6_| (see Methods), calculated from all particle centers. |Ψ_6_| approaches zero for a totally disordered state and reaches unity for a perfect hexagonal crystal. Clearly, at low densities the system is quite disordered with |Ψ_6_| ~ 0.1, however |Ψ_6_| rises sharply around *φ* = 2 and reaches values close to unity at higher densities. Over a broad density range of 5 < *φ* < 20, this highly-ordered structure remains as a universal stable state for all polygon shapes (see Supplementary Note 1 and Supplementary Figs. 5–7 for the other shapes).

Among all three groups, the high-symmetry group behaves almost identical to discs which is well understood. Therefore our study mainly focuses on the intermediate and low symmetry situations, represented by the square and triangle systems, respectively. We directly visualize their high-density ordered state in Fig. 1b, c, with all particle centers connected for a better illustration: clearly the hexagonal crystalline symmetry universally appears in both systems. However, because the orientations of polygons are not identical, this structure is actually a hexagonal plastic crystal. As the rotational symmetry is lowered, the triangular particles tend to produce more 5–7 pair dislocations (labeled in red in Fig. 1c), which make the system more disordered with |Ψ_6_| values smaller than squares. In Fig. 1d, e we plot the 2D Fourier transform of particle centers, which once again confirms a nice hexagonal symmetry in both systems.

Regardless of polygon shapes, the hexagonal plastic crystal becomes a universal stable state over a broad density range. This new phenomenon significantly deviates from short-range interaction systems, in which the stable state depends sensitively on particle shapes. What causes this new phenomenon of shape independency? One simple explanation is that the particles are far from each other instead of in direct contact, and thus the anisotropic effect is too weak to manifest itself. Consequently the particles behave just like the isotropic discs and form the straightforward hexagonal crystal. However, we exclude this trivial possibility by measuring the interaction between polygon pairs aligned with different orientations, and confirm that the interaction is quite anisotropic throughout the density range of the ordered state (see Supplementary Fig. 2).

The true mechanism is revealed by a series of numerical simulations, in which we systematically vary the cut-off distance of interaction. To make a direct comparison, simulations and experiments have the same interaction profiles except that in simulations we set a cut-off distance, *R*_*C*_, beyond which the interaction is identically zero. When *R*_*C*_ is short and particles can only ‘feel’ their nearest neighbors, the stable structures depend sensitively on particle shapes: a square lattice corresponds to square particles and a disordered glassy structure corresponds to triangles, as shown in Fig. 1f at the small cut-off distance *R*_*C* _= 3 (the unit of *R*_*C*_ is the disc particle’s diameter, 6 mm, and also note that triangles do not exhibit the triatic structures observed in ref. ^43^). However, once the cut-off distance reaches large enough value of *R*_*C* _= 6, the hexagonal plastic crystal becomes universally preferred, in both square and triangle systems. This clearly indicates that it is the long-range interaction which prefers large coordination number and produces the universal hexagonal state. Please note that the effective densities in Fig. 1f are *φ* *=* 17.6 for squares and *φ* = 15 for triangles.

We further apply this simulation across a broad density range and summarize all results with two phase diagrams in Fig. 1g, for squares and triangles, respectively. When the cut-off distance *R*_*c*_ is small, the stable states depend on shape and exhibit very poor 6-fold symmetry; however once the cut-off distance is large enough, the hexagonal plastic crystal universally appears. This is true for a broad density range, confirming the robust shape independency experimentally observed. In addition, simulations further reveal that adding or removing the long-range interaction tail only changes the total system energy by a small fraction around 15%, yet it completely eliminates the shape dependency, significantly changes the phase space, and makes the plastic crystal a universal state for various polygons.

### Distinct structures formed by particle vertices

**Fig. 2:**
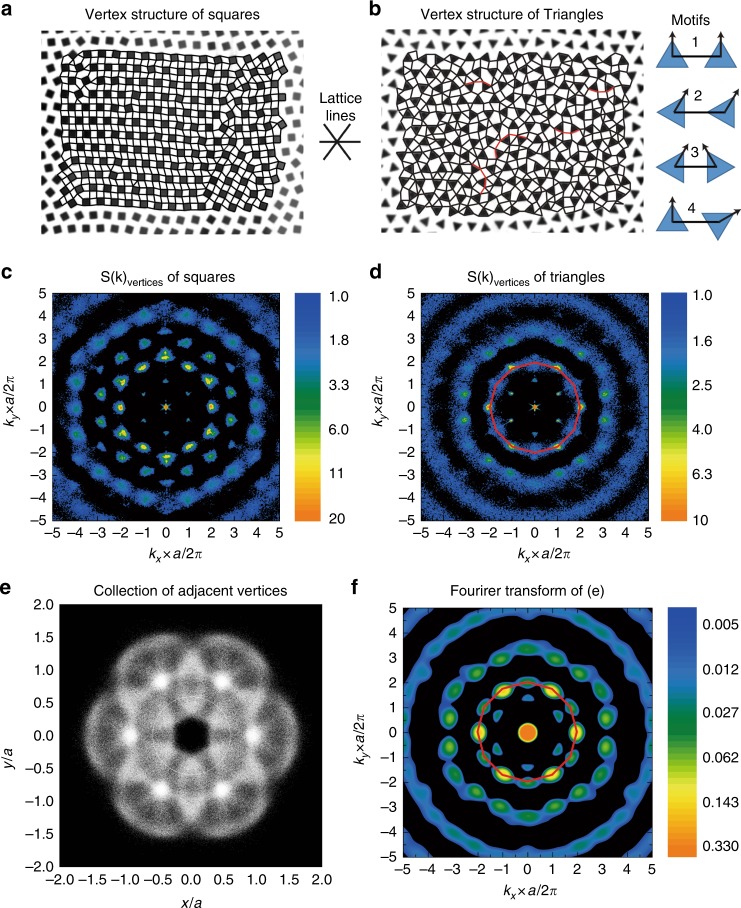
The distinct structures and symmetries of vertices. **a** square vertices form straight stripes aligned with lattice lines. **b** triangle vertices look quite random but the four typical motifs shown on the right are slightly more preferred. The arrow lines in the motifs indicate the particle orientations. The red polylines in the main panel label some typical motif 3 structures which are essential for system relaxation as shown later. Note that Fig. 2a, b use the identical raw images as Fig. 1b, c. **c** 2D Fourier transform of square vertices indicates 6-fold symmetry. **d** 12-fold symmetry appears around *ka*/2*π* = 2 for triangle vertices, with *a* the average particle distance. **e** collection of triangles’ neighboring vertices from all configurations at this density. **f** 2D Fourier transform of **e** also exhibits an obvious 12-fold symmetry around *ka*/2*π* = 2

Experiments and simulations demonstrate that polygon shape makes very slight influence on the static structure of particle centers in the long-range interaction regime. Consequently, we may naturally ask: what is the role of shape in this regime then? Further investigation reveals that shape strongly influences the arrangement of particle vertices and determines the dynamic responses. In Fig. 2a, b we demonstrate the vertex structures of square and triangle systems, with the nearby vertices connected for a better illustration. Strikingly, despite that the raw images are exactly the same as Fig. 1b, c, the patterns formed by vertices give a completely different impression without any obvious hexagonal symmetry.

More specifically, the square vertices in Fig. 2a form straight stripes, due to the fact that most neighboring particles orient in the same direction and align their edges along a certain lattice line. By contrast, triangles form a much more complicated and random pattern, as demonstrated in Fig. 2b. A careful inspection reveals four typical neighboring motifs that are relatively more preferred, as shown in the right panel of Fig. 2b. For motifs 1 and 2, two neighbors have identical orientations indicated by the arrow lines; for motifs 3 and 4, their orientations differ by 60°, which however brings the feature of parallel adjacent edges. This feature gets more and more preferred at higher densities. Statistics further shows that the four motifs are only slightly preferred over other possible patterns, revealing a strong frustration in the vertex arrangement of triangle particles (see Supplementary Note 2 and Supplementary Fig. 8).

To directly visualize the symmetry of vertices, we plot their 2D Fourier transform in Fig. 2c, d. The square vertices exhibit a strong 6-fold symmetry, due to their good alignment with the hexagonal lattice lines. However, an unusual feature appears in the triangle system: the second shell around *ka*/2*π* = 2 (*a* is the average particle distance) looks no longer like a hexagon but more like a dodecagon, which suggests a 12-fold symmetry. Note that *ka*/2*π* = 2 corresponds to the typical distance between two adjacent vertices.

To probe the origin of this 12-fold symmetry, we collect all possible connections between two adjacent vertices, put one end on a common origin, and plot the other end as one bright spot in Fig. 2e. The brightness of this image thus directly reflects the probability density of adjacent vertices collected from all configurations, and the image exhibits an obvious 6-fold symmetry. However, once the image is Fourier transformed into *k* space in Fig. 2f, a 12-fold symmetry clearly appears around *ka*/2*π* = 2. This feature is quite similar to the one in Fig. 2d and thus explains its origin: for triangle particles the neighboring vertices arrange cooperatively to form 2 sets of 6-fold symmetry, which combine coherently and produce a 12-fold symmetry. More experiments further reveal that such a cooperation increases with density: the feature grows more and more pronounced in the density range of 13 < *φ* < 20 but becomes weaker below *φ* ~ 13 (see Supplementary Note 5 and Supplementary Fig. 13).

### Particle shape determines system relaxation

**Fig. 3:**
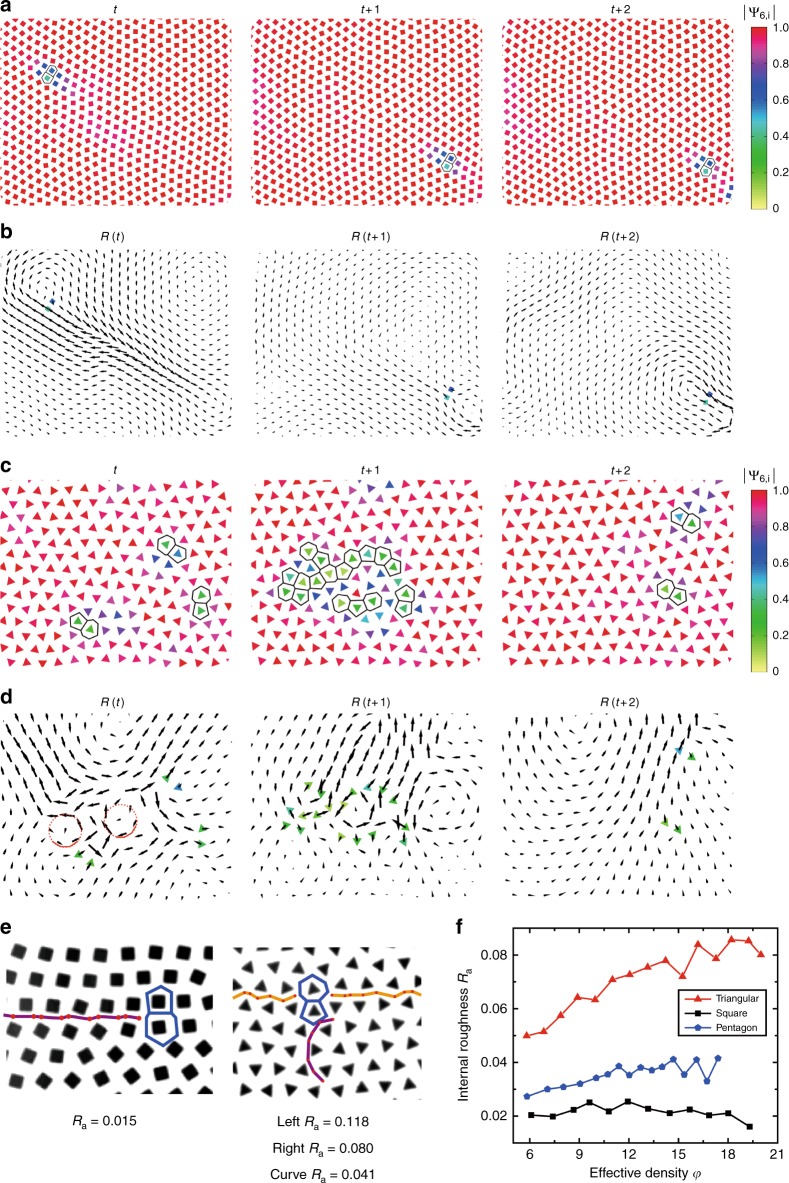
Relaxation dynamics determined by vertex structures. **a** the square system relaxes through dislocation gliding. The 5–7 pair is labeled by two polygons and the particle color represents order parameter. **b** The displacement field, R(*t*), for the gliding event in **a**. R(*t*) represents the displacement from frame *t* to *t*  + 1 and large displacements concentrate along the gliding line. Colored particles indicate the 5–7 pair. **c** the triangle system relaxes via defect loops. **d** the displacement field of the relaxation event in **c**. Colored particles indicate defects. The red circuits label motif 3 structures which highly correlate with the excitation field and provide a structural basis for defect loop. **e** the internal roughness, *R*_*a*_, is small in square system but much larger in triangle system. However *R*_*a*_ reduces significantly along a nearby motif 3 structure. The two polygons indicate the 5–7 pair dislocation. **f** average *R*_*a*_ along all lattice lines in triangle, square, and pentagon systems

Apparently different particle shapes produce different vertex structures, but how does this difference affect system properties? We demonstrate that it influences the basic relaxation dynamics and makes systems respond distinctively under external perturbations. Because ordered structures normally relax through defect dynamics, we thus probe the movements of defects, typically 5–7 pair dislocations, at single-particle level (see Supplementary Movie 1). In Fig. 3a–d, we illustrate the typical relaxation dynamics in the same samples as the ones shown in Figs. 1 and 2. In the square system of Fig. 3a, we first identify a typical 5–7 pair dislocation as labeled by the two polygons, and then analyze its motion during three consecutive frames. The color of each particle represents its local order parameter |Ψ_6,*i*_| and clearly particles are poorly ordered around the dislocation. During the three frames, the dislocation pair first moves a large and then a small step in frame *t* + 1 and *t* + 2, respectively, along the same lattice line. This relaxation is a typical dislocation gliding event.

To illustrate the underlying dynamics of this gliding, we plot the displacement field of every particle in Fig. 3b (also see Supplementary Movie 2), with the dislocation pair labeled by two colored squares. The field R(*t*) represents the displacement field between frame *t* and *t* + 1. Clearly large displacements occur around the same lattice line of the dislocation gliding, suggesting that a local excitation along this line produces the gliding event. Moreover, the features of global swirls and large displacements agree well with the soft modes calculated from covariance matrix (see Supplementary Figs. 14a and 15a), indicating that the soft modes produced by soft defect spots are responsible for this excitation (see Supplementary Note 6). This result agrees well with the previous discoveries in colloidal and hard granular systems^50–52^, except that we now expand this powerful soft-mode mechanism into anisotropic and long-range interacting systems.

In contrast to the square system, totally different relaxation dynamics appears in the triangle system: dislocation gliding no longer appears in Fig. 3c; instead a loop of defects forms and the system relaxes through the creation and annihilation of this loop (also see Supplementary Movie 3). Such kind of defect-loop relaxation typically appears in quasicrystals^44^ but surprisingly it becomes the major relaxation approach in our triangle systems.

To uncover the mechanism of this relaxation, we again plot the displacement field in Fig. 3d and find large displacements near the defect loop. Similar as the square system, there is a good agreement between the displacement field and the soft modes (see Supplementary Fig. 15b), indicating the soft modes as the underlying relaxation mechanism. More interestingly, a careful inspection further reveals that motif 3 structures frequently appear near the defect loops, and correlate closely with the loops’ swirling displacement field, as indicated by the red circuits in Fig. 3d (the solid parts indicate motif 3). This finding suggests motif 3 as a structural basis for the defect loop relaxation.

Why the motif 3 structure is so special for defect loop relaxation? More fundamentally, at the particle level what controls relaxation behaviors and produces two distinct relaxation mechanisms, i.e., dislocation gliding vs. defect loop? We illustrate this fundamental issue by defining a microscopic parameter, the ‘internal roughness’, *R*_*a*_, at single-particle level. As shown in Fig. 3e: we first identify the midpoints of neighboring vertices, and then link these midpoints to form a zigzag line. The standard deviation, *σ*, of this zigzag line describes the internal roughness produced by particle vertices. We further renormalize *σ* with the average particle distance *a* and obtain the dimensionless internal roughness, *R*_*a* _= *σ*/*a*. Because *R*_*a*_ is dimensionless, it can be generally defined and applied in various systems beyond our model system.

As shown in the left panel of Fig. 3e, squares apparently produce a very small roughness around *R*_*a* _= 0.015. By contrast, triangles generate a much larger roughness, *R*_*a* _= 0.08 to 0.1, as illustrated in the right panel. However, *R*_*a*_ reduces significantly (over 50%) to 0.04 along a curved path nearby, which is exactly along a motif 3 structure and a defect loop forms right at this location. Therefore, our data suggest that motif 3 structures can effectively reduce *R*_*a*_, facilitate defect movements along them, and provide a structural basis for defect loop relaxation.

To summarize, our results suggest that small internal roughness is essential for the movements of dislocations. When *R*_*a*_ is small such as in square systems, dislocations tend to glide along lattice lines; however, when *R*_*a*_ is too large for dislocations to glide along (such as in triangle systems), the structure will have to relax through defect loops. Even the formation of defect loops requires small roughness, which is realized by special structures such as the motif 3 structures. This internal-roughness picture successfully explains the two observed relaxation mechanisms at single-particle level.

To test the validity and robustness of this picture, across a broad density range we measure the internal roughness of triangle, square, and pentagon systems. We compare their system’s overall *R*_*a*_ averaged over all lattice lines in Fig. 3f: the square and pentagon systems are relatively smooth (i.e., with small *R*_*a*_) and thus relax through dislocation glidings; however the triangle systems are quite rough and mainly relax via defect loops. Moreover, our data further suggest a critical roughness value, *R*_*a* _~ 0.04, below which glidings dominate and above which defect loops take over. Even for large-roughness systems that relax via defect loops, we still need the special structure of motif 3 to make *R*_*a* _~ 0.04, which enables the relaxation to proceed. Based on these observations, we propose that making *R*_*a*_ below a critical value could be a general requirement for plastic relaxations, although the specific critical value may vary with system and condition.

## Discussion

In conclusion, we have experimentally explored the new regime of packing long-range interacting and anisotropic building blocks. Although particles have various polygonal shapes, different systems however reach a universal state, the hexagonal plastic crystal. This state originates from the long-range interaction going beyond the nearest neighbors. Despite its slight influence on the static structure, the particle shape strongly affects the system internal roughness, and determines the relaxation dynamics. Our data further suggest that defects prefer to move along small-roughness paths, and may only relax under a certain critical roughness regardless of in dislocation gliding or defect loop relaxation. This finding may provide a fundamental picture for the relaxation of plastic crystals, which widely appear in square ice and spin-frustrated lattices^53^. It could even extend to more disordered systems such as metallic glasses, and help to explain their high rigidity and low plasticity: because of the really large *R*_*a*_ values throughout the glassy system, the soft defect spots in metallic glasses are essentially locked in space and very difficult to move and relax, either via dislocation gliding or defect loop. Therefore, our analysis may help to explain why disordered metallic glasses can achieve higher rigidity and lower plasticity than their perfectly-ordered crystalline counterparts.

## Methods

### Experimental details

Our system is made up of Nd_2_Fe_14_B magnetic particles with various shapes: disc, hexagon, pentagon, square and triangle, and all particles are coated with a smooth nickel layer. We show the top and side views of the particles in Supplementary Fig. 1. In the *x*–*y* plane, all polygons have a center-to-vertex distance 3.00 ± 0.03 mm and the discs have a radius of 3.00 ± 0.03 mm; in the *z* direction, all particles have the thickness 2.97 ± 0.03 mm. The particles have a strong permanent magnetization along the *z* direction, with a maximum magnetic field *B* ~ 0.5 *T* at the magnetic poles. In the *x*-*y* plane, the anisotropic magnetic field can extend its influence to large distances of multiple particle size. Using a force sensor, we can directly measure the repulsive interaction force between two particles, with different relative orientations (e.g., edge-to-edge, edge-to-tip, and tip-to-tip), as shown in Supplementary Fig. 2a, b for square and triangle particles, respectively. The force profile is close to a power-law, *F* ~ *r*^−4.2^ (or an interaction potential *U*(*r*) ~ *r*^−3.2^). Significant anisotropy can be observed at small and medium *r*. With these magnetic particles, we can study single-particle behaviors in systems with long-range and anisotropic interactions.

We confine the particles between two glass plates to form a single-layer 2D system. The thickness of the spacer is carefully controlled at 3.12 ± 0.04 mm, which ensures that the particles do not tilt significantly (the tilting angle is less than 5 degrees) in all experiments. To provide external excitations, we use a plate with randomly pinned magnets slowly moving back and forth under the 2D system, with an amplitude A = 420 mm and period *T* = 4.9 *s*, as illustrated in Supplementary Fig. 3 and Supplementary Movie 1. The perturbation strength can be controlled by the distance *H* between the perturbation plate and the system confining box. After each set of perturbations (50 cycles), the perturbation plate is removed, and the system relaxes into a new mechanical equilibrium configuration, which sits at one specific local minimum of the potential energy landscape. We record plenty of such local minima configurations (typically more than 500) and probe their transition kinetics.

The friction coefficient in our system is quite small, 0.15 ± 0.03. Thus the repulsive force between two particles can overcome the friction at very large distances, typically above 10 particle radii. Therefore, for each shape we define an effective radius, *r*_*e*_, as the exact distance at which the repulsive force matches the friction and two particles start to ‘feel’ each other. Based on *r*_*e*_, we then define the effective density, $\varphi = N\pi r_e^2/S$, with *N* the particle number and *S* the total area of the system. This definition essentially treats each particle as a soft disc with the radius *r*_*e*_ and at *φ* = 1 they start to feel each other. Because of the long-range interaction, we can vary the effective density *φ* over a broad range from 1 to about 20 (particle number from about 100 to 2000), throughout which particles can still feel and interact with each other. This has rarely been achieved for short-range interactions. We average the order parameters and correlation functions over 20 to 25 independent experiments, and the particles at boundary are not included in the results. Some typical configurations from low to high densities are illustrated in Supplementary Fig. 4, with the top row showing the square systems and the bottom row showing the triangle systems. Note that the confining box is hexagonal, which minimizes the boundary effect for a hexagonal plastic crystal (See Supplementary Note 4 and Supplementary Fig. 12 for the discussion of boundary effect).

To obtain a systematic understanding, we gradually break up the rotational symmetry and perform extensive experiments on disc, hexagon, pentagon, square and triangle systems. Their behaviors fall into three categories: high-symmetry (disc and hexagon), intermediate-symmetry (pentagon and square) and low-symmetry (triangle). The high-symmetry situation is well understood and our study focuses on the intermediate and low symmetries. In the main text, we mainly show squares and triangles. For other shapes, hexagons behave almost identical to discs which are well understood, and the pentagon system is similar to the square system but with larger frustrations. The results of pentagon system are shown in Supplementary Note 3 and Supplementary Figs. 9–11.

### Calculation of |Ψ_6_|

The global bond orientational order |Ψ_6_| is defined as $|\Psi _6| = \left\langle {\mathop {\sum}\nolimits_i {\psi _{6,i}} } \right\rangle$, where $\psi _{6,i} = \frac{1}{{N_i}}\mathop {\sum}\nolimits_j {e^{i6\alpha _{ij}}}$ with *α*_*ij*_ being the angle of the *j*_*th*_ bond with respect to the *x* axis and *N*_*i*_ the number of neighbors of particle *i*.

## Supplementary information

Supplementary Information

Description of Additional Supplementary Files

Supplementary Movie 1

Supplementary Movie 2

Supplementary Movie 3

## Data Availability

The primary data that support the findings of this study are available from the corresponding authors upon request (Email: xuleixu@cuhk.edu.hk or tanpeng@fudan.edu.cn).

## Code availability

The primary code that support the findings of this study are available from the corresponding authors upon request (Email: xuleixu@cuhk.edu.hk or tanpeng@fudan.edu.cn).
