# Supplementary material for: A universal state and its relaxation mechanisms of long-range interacting polygons
Source: Nat Commun. 2019 Apr 15;10:1737. doi: 10.1038/s41467-019-09795-6 (PMC6465257; doi:10.1038/s41467-019-09795-6)
Supplement: Supplementary file 1 — Supplementary Information [file 41467_2019_9795_MOESM1_ESM.pdf]

Supplementary Information for
"A universal state and its relaxation mechanisms of long-range interacting polygons"

Shen et al

Supplementary Figure 1

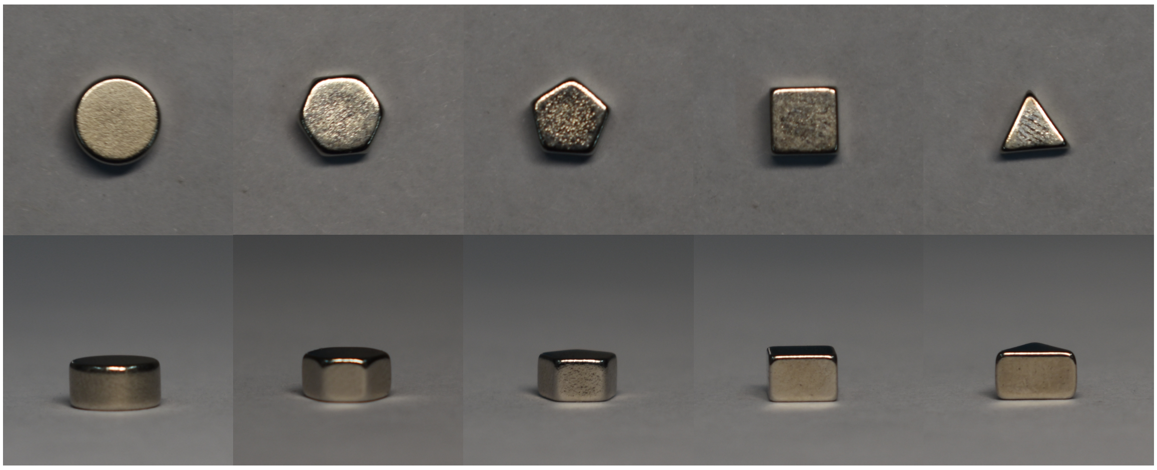

Supplementary Figure 1: Illustration of the disc, hexagon, pentagon, square and triangle magnets. The first row is the top view (x-y plane) and the second row is the side view. The magnetization is along the z-axis, perpendicular to the $x-y$ plane shown in first row.

Supplementary Figure 2

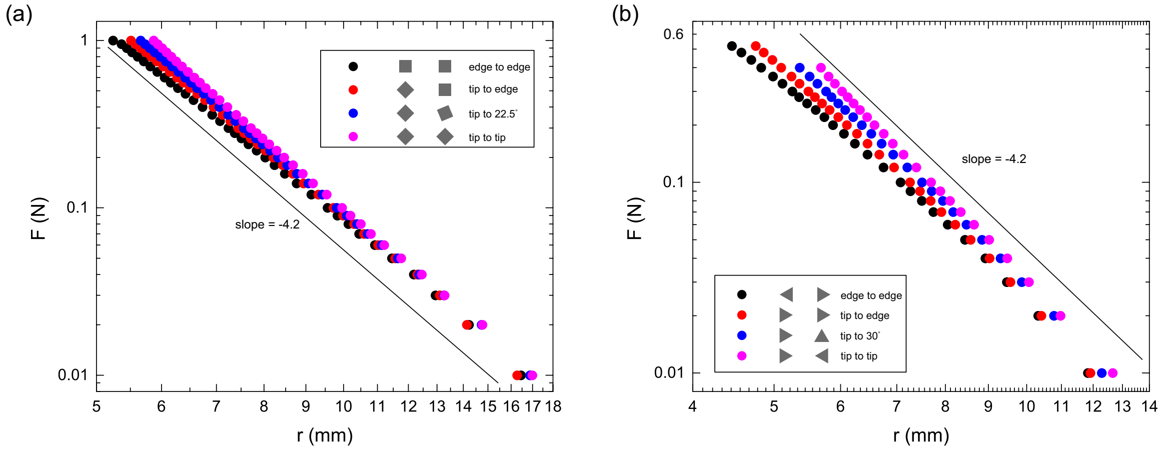

Supplementary Figure 2: (a) Repulsive force profiles between two square particles at various center-to-center distance r. Different curves correspond to different relative orientations. (b) The same measurements between two triangle particles. In both situations, the forces are strongest in the tip-to-tip configuration and the weakest in the edge-to-edge configuration at same r. The force profiles are close to a power-law dependence on r with the power around -4.2, which is shown by the black line.

Supplementary Figure 3

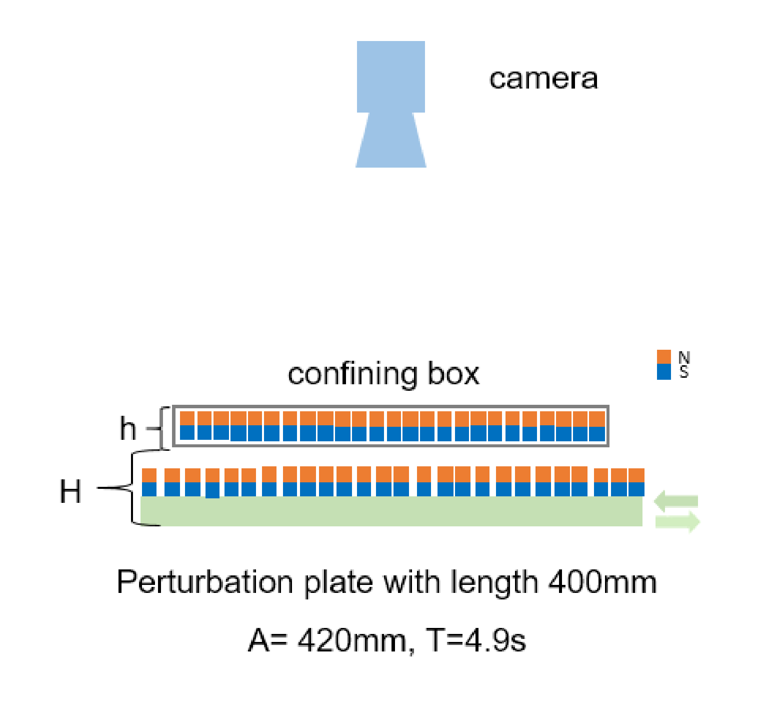

Supplementary Figure 3: The schematic illustration of the experimental setup. We use two glass plates and a spacer to form the confining box. The spacer thickness is carefully controlled at $3.12 \pm 0.04mm$ to ensure that particles do not tilt significantly. Under the confining box, a $400mm$ perturbation plate formed by randomly pinned magnets moves back and forth periodically to provide the external excitation. Its moving amplitude is $A = 420mm$ and time period is $T = 4.9s$. We can control the perturbation strength by tuning the distance H. After every 50 cycles of perturbations, we record the configuration with a CMOS camera. The orange and blue colors represent the north and south poles of magnets respectively.

Supplementary Figure 4

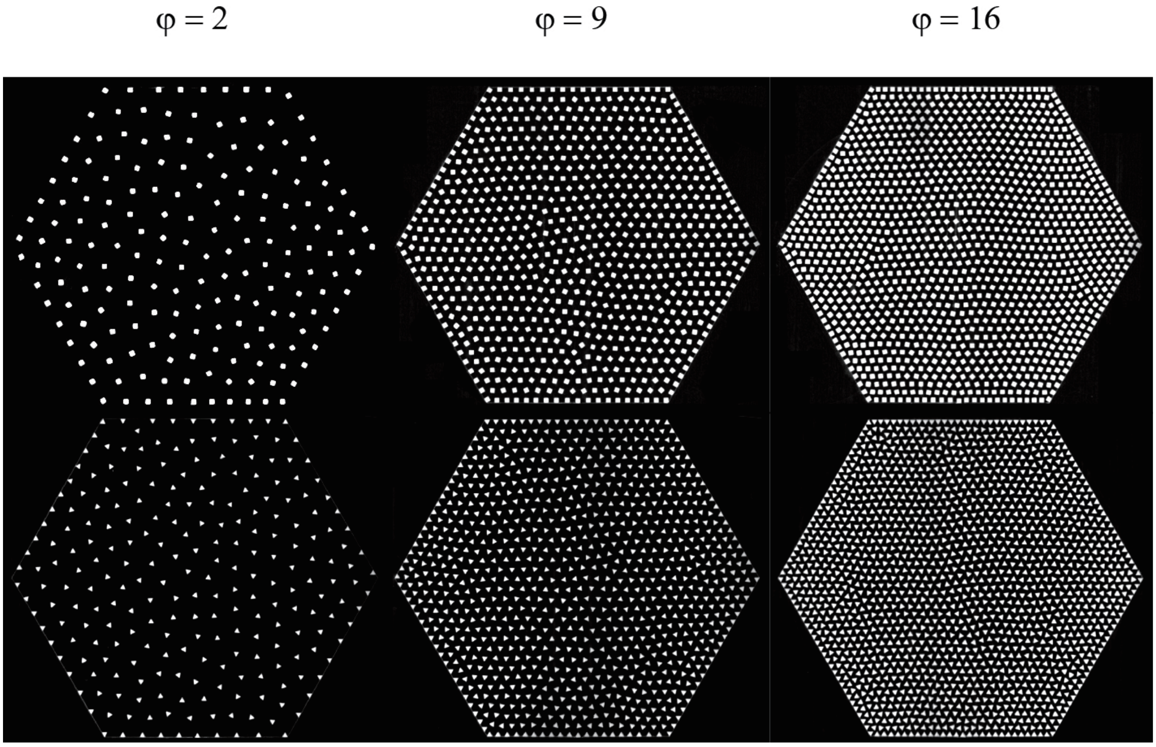

Supplementary Figure 4: Configurations of square-particle system (Top) and triangle-particle system (Bottom) at three typical densities. At low densities ($\varphi = 2$), the structures are disordered. At intermediate ($\varphi = 9$) and high densities ($\varphi = 16$), the particles are organized into ordered complex structures.

Supplementary Note 1: bond orientational and translational orders of particle centers

Similar to the results in Fig.1a, Supplementary Figure 5 gives the global bond orientational order parameter for the hexagon and pentagon systems. Clearly at low densities the structure is disordered but an ordered hexagonal state appears at high densities, consistent with the other shapes shown in Fig.1a.

To further characterize the bond orientational correlation of the particle centers, we calculate $g_6(r)/g(r)$. Here $g_6(r) = \langle \psi_6(\mathbf{r}_i)\psi_6^*(\mathbf{r}_j)\delta(r - |\mathbf{r}_i - \mathbf{r}_j|)\rangle$ is the spatial correlation of hexatic order. $\psi_6(\mathbf{r}_i) = \frac{1}{N_i}\sum_j e^{i6\alpha_{ij}}$ with α_{ij} being the angle of the j_{th} bond with respect to the x axis of particle i and N_i the number of neighbors. $g(r) = \langle \delta(r - |\mathbf{r}_i - \mathbf{r}_j|)\rangle$ is the pair correlation function. $\langle\rangle$ indicates the average over different particles and configurations.

Supplementary Figure 6 shows the bond orientational correlation, $g_6(r)/g(r)$, of discs, squares and triangles at different densities. In the density range of $7 < \varphi < 20$, which corresponds to the ordered plateau of $|\Psi_6|$ in Fig.1a of main text, all three systems show a quasi-long-range bond orientational correlation, with a good power law decay of $g_6(r)/g(r)$. The decay power, which describes the correlation of the bond orientational order, reveals that discs have the best bond orientational order and triangles are the worst, consistent with

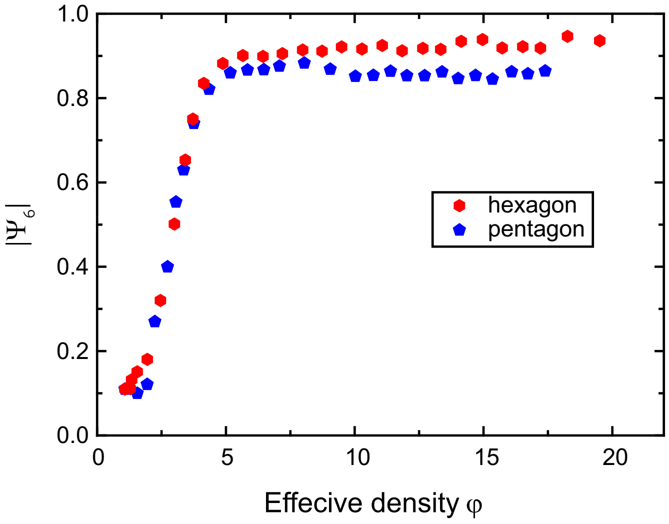

Supplementary Figure 5: The global bond orientational order $|\Psi_6|$ at different densities for the hexagon and pentagon centers. The structure is quite disordered at low densities but an ordered state appears at high densities, similar to Fig.1a in the main text.

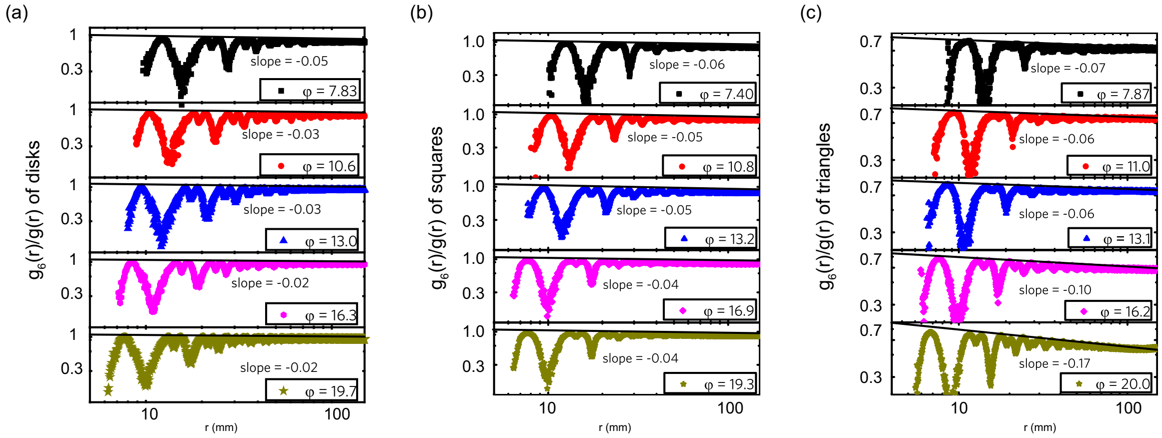

Supplementary Figure 6: $g_6(r)/g(r)$ for disk system (a), square system (b) and triangle system (c) at different densities. $g_6(r)/g(r)$ shows power law decay with power index close to zero, which means a quasi-long range bond orientational order in system.

their $|\Psi_6|$ values. Clearly a stronger anisotropy in the interaction leads to a larger lattice distortion.

In Supplementary Figure 7, we show $g(r) - 1$ to compare the translational correlation of the three systems. We find larger $g(r) - 1$ values in more symmetric systems (disk>square>triangle). Interestingly, different from the bond orientational correlation, $g(r) - 1$ in all three systems has a similar power-law decay with power index around -2.0, in the density range of $7.4 < \varphi < 20.0$.

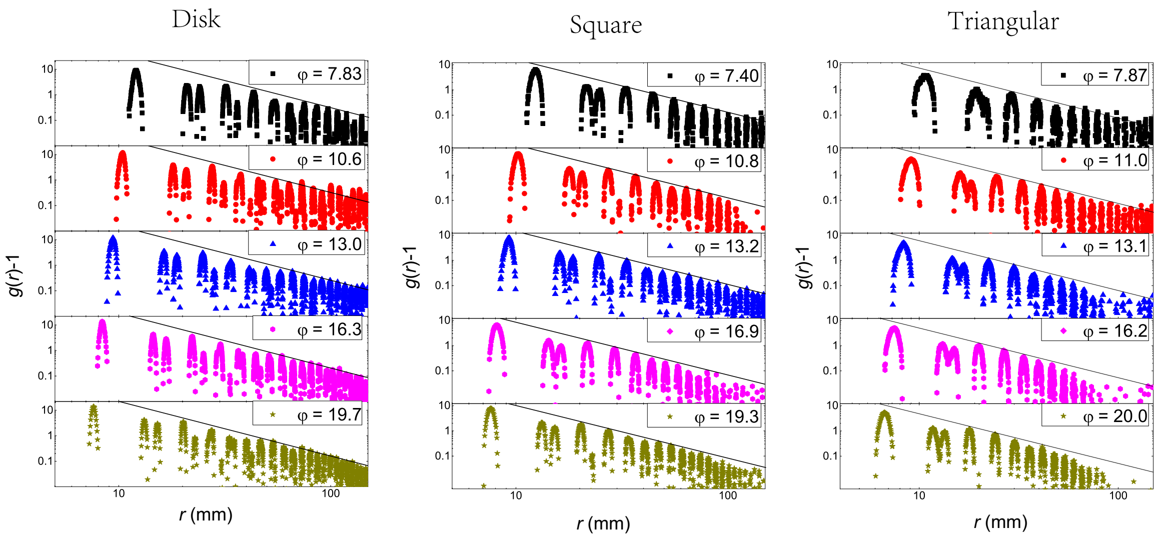

Supplementary Figure 7: $g(r) - 1$ of disk-, square- and triangle-particle systems. In all conditions, the translational correlation shows a similar power-law decay, $g(r) - 1 \sim r^{-2}$, as indicated by the black lines.

Supplementary Note 2: relative orientation between neighboring square and triangle particles

To clarify the factor of shape in vertex arrangement, we measure the relative particle orientation as a representative quantity because it is closely related to particle shape. We measure the relative orientations between neighbors and plot the statistics of these relative angles in square and triangle systems respectively. As shown in Supplementary Figure 8, the squares' relative orientations between neighbors are favored at 0° and 90°, which are equivalent due to the 4-fold symmetry (all angles above 90° need to mod(90°)). The probability at 0° (or equivalently 90°) can reach one order of magnitude higher than other relative angles at high densities. This indicates a strong preference of neighboring squares to align into the same orientation along lattice lines.

However, for triangles in the second row, there exist three preferences, locating at 0°, 60° and 120° respectively (note that 0° and 120° are equivalent due to the 3-fold symmetry and all angles above 120° need to mod(120°)). All these preferred probabilities are only slightly higher than other relative angles, indicating many orientation possibilities and strong frustrations in the arrangement of triangle vertices. Note that parallel adjacent edges with the relative angle of 60° (motifs 3 and 4) are more and more preferred at higher densities.

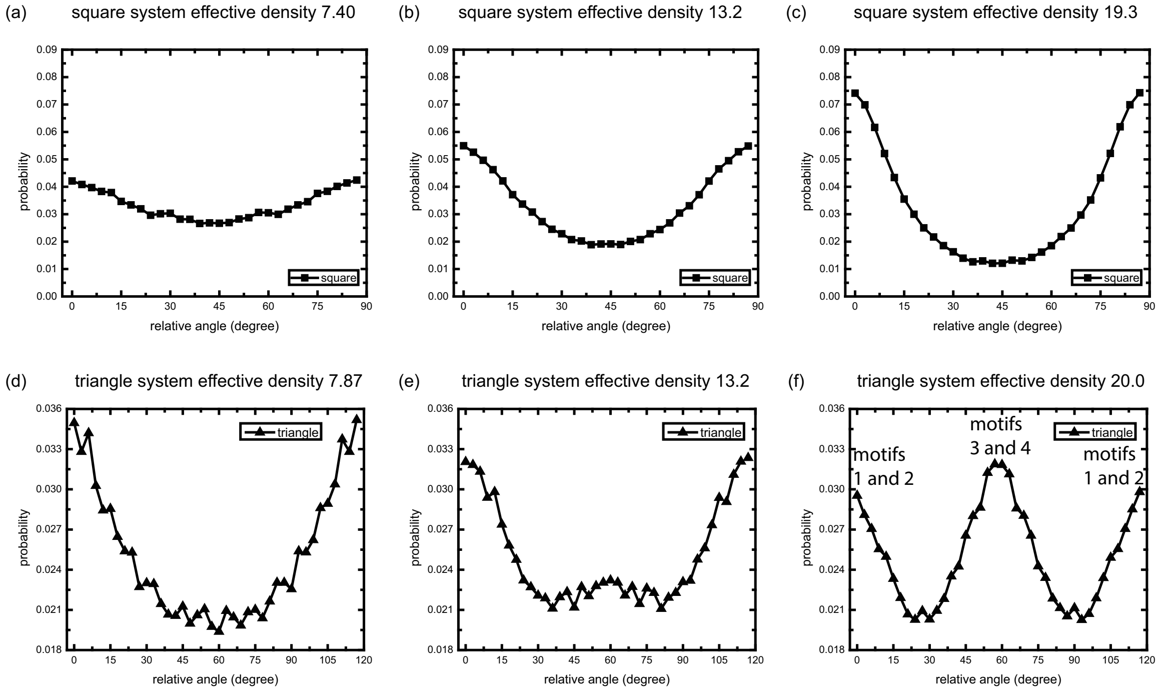

Supplementary Figure 8: Distribution of relative orientational angle of neighboring particles at low, medium, and high densities. Because of rotational symmetry m of the shaped particles, the angles are in the range $[0°, 360°/m]$. Upper, (a)-(c), square-particle system with $m = 4$. Lower, (d)-(f), triangle-particle system with $m = 3$.

Supplementary Note 3: pentagon-particle systems

As mentioned in the main text, different particle shapes fall into three categories, high-symmetry (disc and hexagon), intermediate-symmetry (pentagon and square), and low-symmetry (triangle). Because the behaviors of high-symmetry particles such as discs are well understood, we focus on the intermediate- and low-symmetry situations (i.e., pentagons, squares and triangles). Since the squares and triangles are illustrated in the main text, this section mainly demonstrates the data of pentagons. We will see that the behaviors of pentagons are similar to squares, and thus the two shapes form the intermediate-symmetry category.

The static structural factor $S(\mathbf{k})$ of both particle centers and vertices at $\varphi = 16.1$ are shown in Supplementary Figure 9. $S(\mathbf{k})_{\text{centers}}$ shows a six-fold global symmetry of the particle-center positions. Comparing with $S(\mathbf{k})_{\text{vertices}}$ of the square-particle system (Fig.2c) which shows four non-uniform shells, $S(\mathbf{k})_{\text{vertices}}$ of pentagons gives three non-uniform shells.

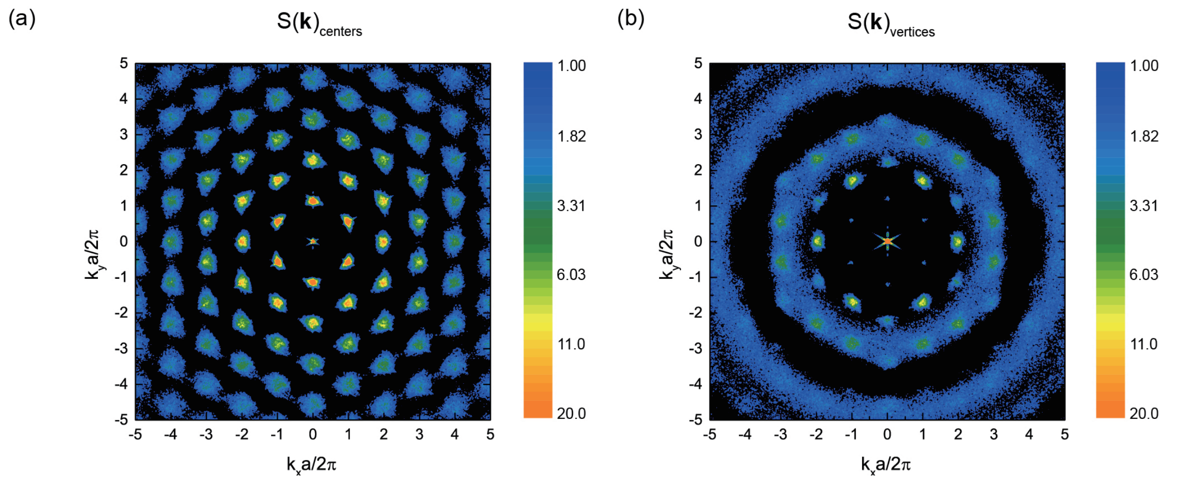

Supplementary Figure 9: Static structure factor of the pentagon-particle system at $\varphi = 16.1$. (a) $S(\mathbf{k})_{\text{centers}}$ for particle centers. (b) $S(\mathbf{k})_{\text{vertices}}$ for particle vertices.

Therefore, the symmetry mismatch between particle shape and the bulk structure are more significant in pentagon-particle systems than in square-particle systems.

The orientations of the pentagons still tend to align with the lattice lines, similar to the square situation. We can observe this alignment with the relative orientation data in Supplementary Figure 10, which shows the preference of relative angle at $0°$ (or equivalently $72°$). But the preference of alignment is relatively weaker compared with the square data at the same density. This weak alignment forms short-range smooth stripes, which also make the dislocation gliding to be the main relaxation mechanism in pentagon systems, similar to the square systems. One typical dislocation gliding event in pentagon systems is shown in Supplementary Figure 11.

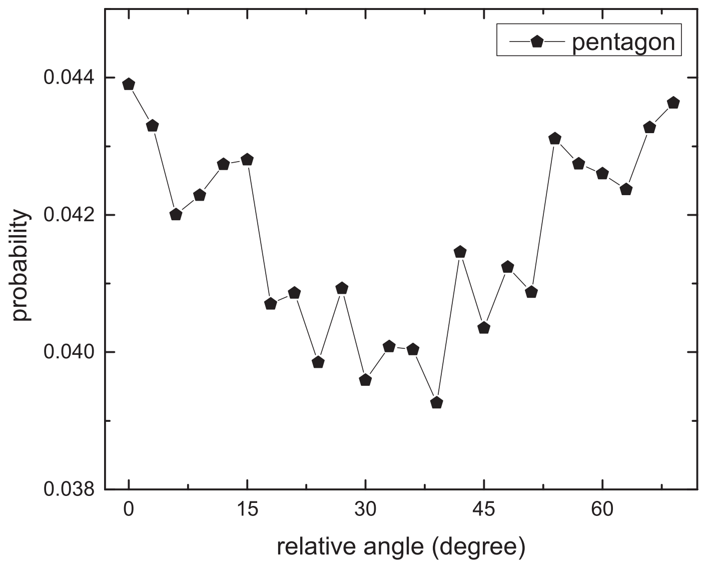

Supplementary Figure 10: Distribution of relative angle of neighboring pentagon particles at $\varphi = 16.1$. The main peak is at 0° (72°), indicating a tendency of the neighboring pentagons to align in parallel.

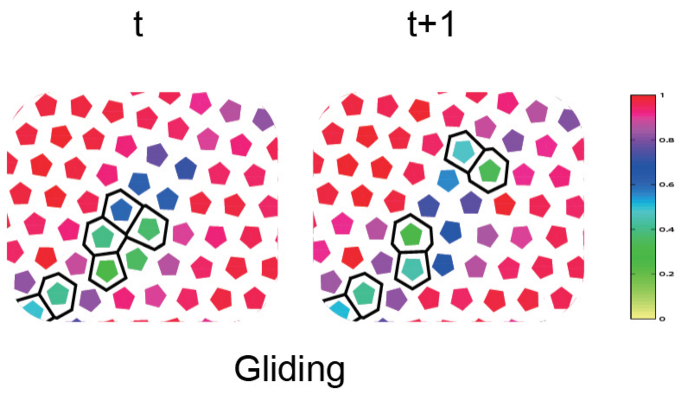

Supplementary Figure 11: Typical dislocation gliding dynamics in a pentagon-particle system at density $\varphi = 16.1$. The colors represent $|\psi_{6,i}|$ values and the polygons from Voronoi tessellation label the defects with 5 or 7 neighbors.

Supplementary Note 4: boundary effects

Our data shown in the main text are inside a hexagonal confining box, as illustrated in Supplementary Figure 4. Besides the hexagonal boundary, we also performed measurements with other boundary conditions, such as the square boundary and the circular boundary. We find that the results are essentially the same with different boundary conditions, demonstrat-

ing the robustness of our findings which are independent of boundary conditions. However, hexagonal boundary is the best to avoid symmetry mismatch between the boundary and the bulk structures, and other types of boundaries tend to induce more boundary effects. We show one typical boundary effect from a square boundary in Supplementary Figure 12: Two grain boundaries starting from the square boundary cut the bulk structure into three grains. But even with such a strong boundary effect, the particle centers clearly form a six-fold symmetric structure, consistent with the data shown in the main text. Therefore, all the behaviors shown in the main text are intrinsic properties instead of caused by any specific boundary condition.

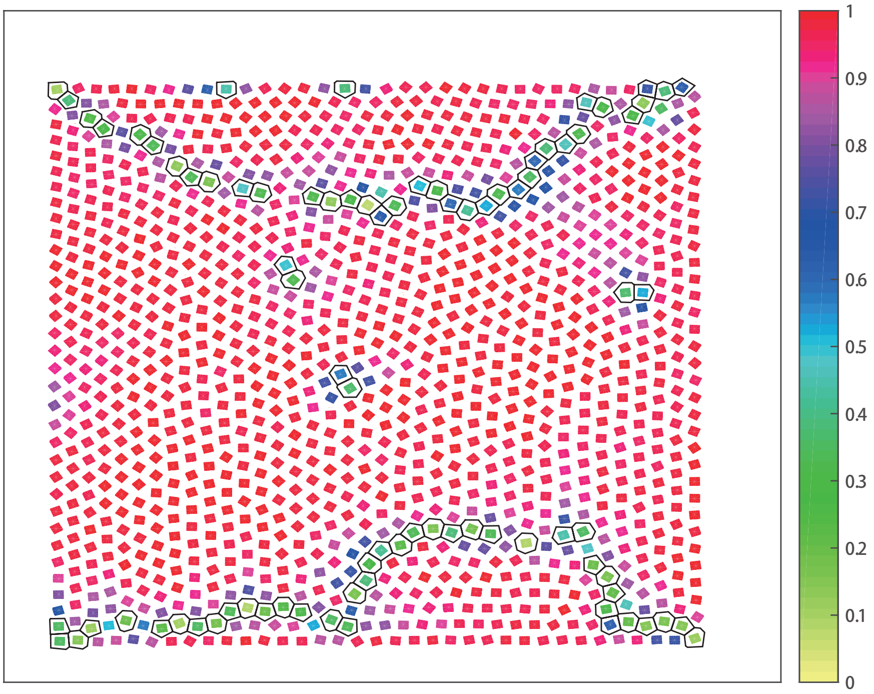

Supplementary Figure 12: Configuration of a square-particle system inside a square confining box at $\varphi = 16.6$. The colors represent $|\psi_{6,i}|$ values and the polygons from Voronoi tessellation label the defects with 5 or 7 neighbors. Two grain boundaries starting from corners cut the structure into three grains. Except for the grain boundaries, the system has good hexagonal bond orientational order.

Supplementary Note 5: 12-fold symmetry by adjacent vertices in triangle systems

Besides the typical example shown in Fig.2f in the main text, here we systematically illustrate the Fourier transformation of all line segments connecting adjacent vertices of triangle particles in Supplementary Figure 13: from low to high densities, the 12-fold symmetric feature around $k = 2$ becomes more and more pronounced. At lower densities, the anisotropy of the particle is weaker, and the neighboring particles interact with each other in a more isotropic way. The particles can rotate more freely at lower densities and the neighboring particles do not align well with parallel adjacent edges. The relative particle orientation distribution is more random and the cooperation which gives the 12-fold symmetry is also negligible. Thus the robust feature in the Fourier transformation is the 6-fold lattice symmetry. At high densities above $\varphi \sim 13$, more and more particle pairs align with parallel adjacent edges, and such a cooperation produces the amazing 12-fold symmetry.

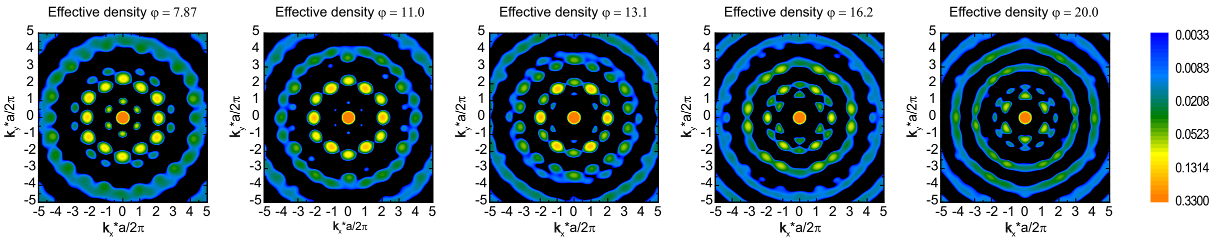

Supplementary Figure 13: Fourier transformation of all line segments connecting adjacent vertices in triangle systems. As the density increases from left to right, the 12-fold symmetric feature around $k = 2$ becomes more and more pronounced.

Supplementary Note 6: soft modes drive system relaxations

Both types of rearrangement behaviors, the dislocation gliding (squares, pentagons, hexagons, discs) and the defect loop (triangles), are highly correlated to the low-frequency soft modes and can be understood with the soft spot picture. Using the particle displacements before each rearrangement event, we can extract the low-frequency eigenmodes (i.e., soft modes) of the system with covariance matrix method. From these quasi-localized low-frequency modes (i.e., soft modes) shown in Supplementary Figure 14, we can easily identify places where particles can explore larger regions nearby, which define the soft spots indi-

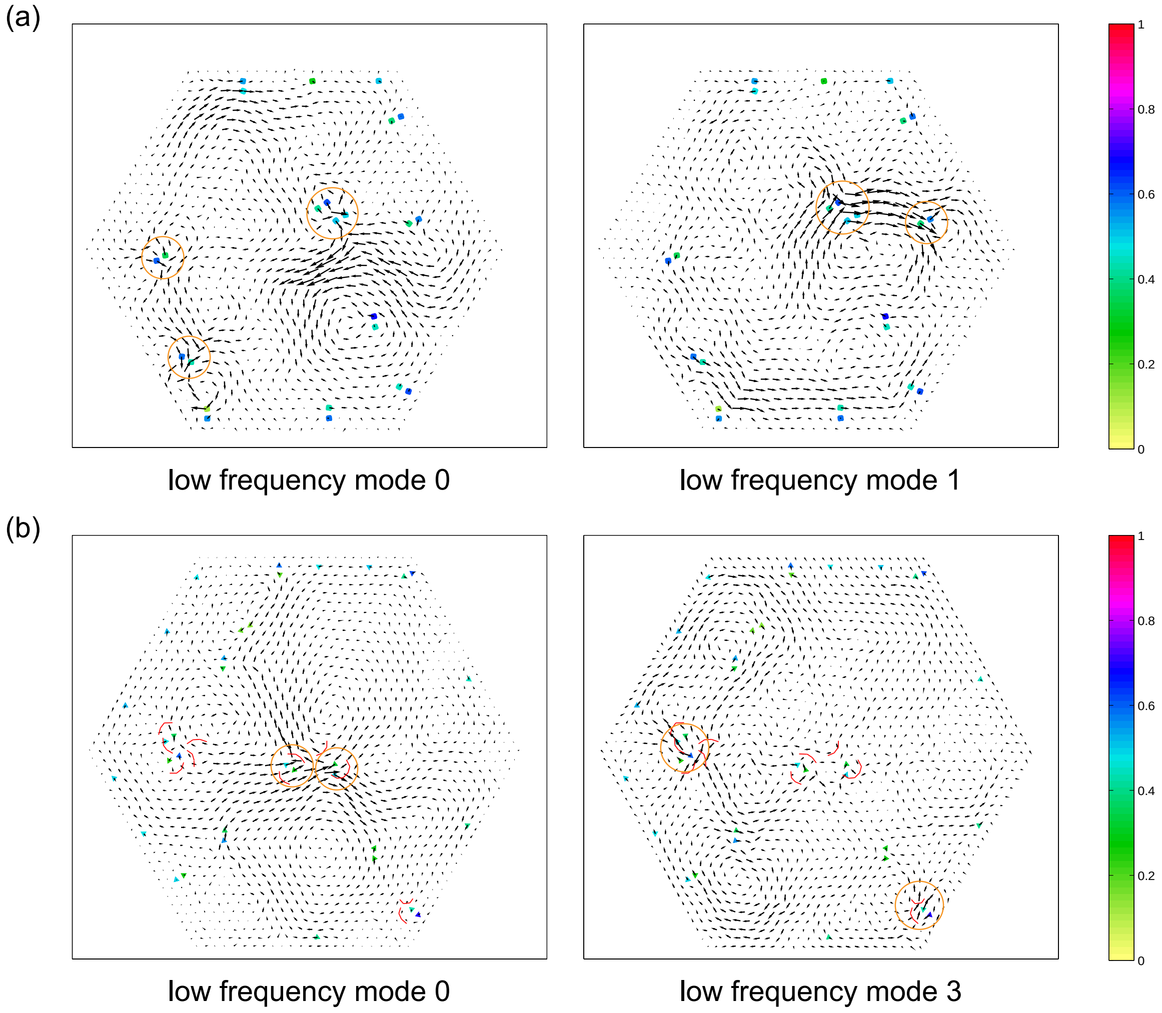

Supplementary Figure 14: Some typical low-frequency soft modes in (a) square and (b) triangle systems. These modes demonstrate the collective motions at low frequencies (i.e., low energy levels). The regions with large displacements are soft spots which we label with orange circles. The soft spots overlap well with dislocations. Note that in different soft modes the soft spots can be different. In triangle systems, the motif 3 structures (red poly-lines) frequently appear around dislocations and soft spots, providing a structural basis for defect loop relaxations.

cated by the orange circles. Apparently, these orange circles overlap with some particular dislocations, indicating a high correlation between soft spots and dislocations. Because the soft-spot mechanism indicates that system relaxes via the excitation of soft modes mainly through large movements at soft spots, it agrees well with our observation of large excitations around dislocations. Moreover, in the triangle system we label the motif 3 structures as red poly-lines, which frequently appear near the dislocations and provide a structural

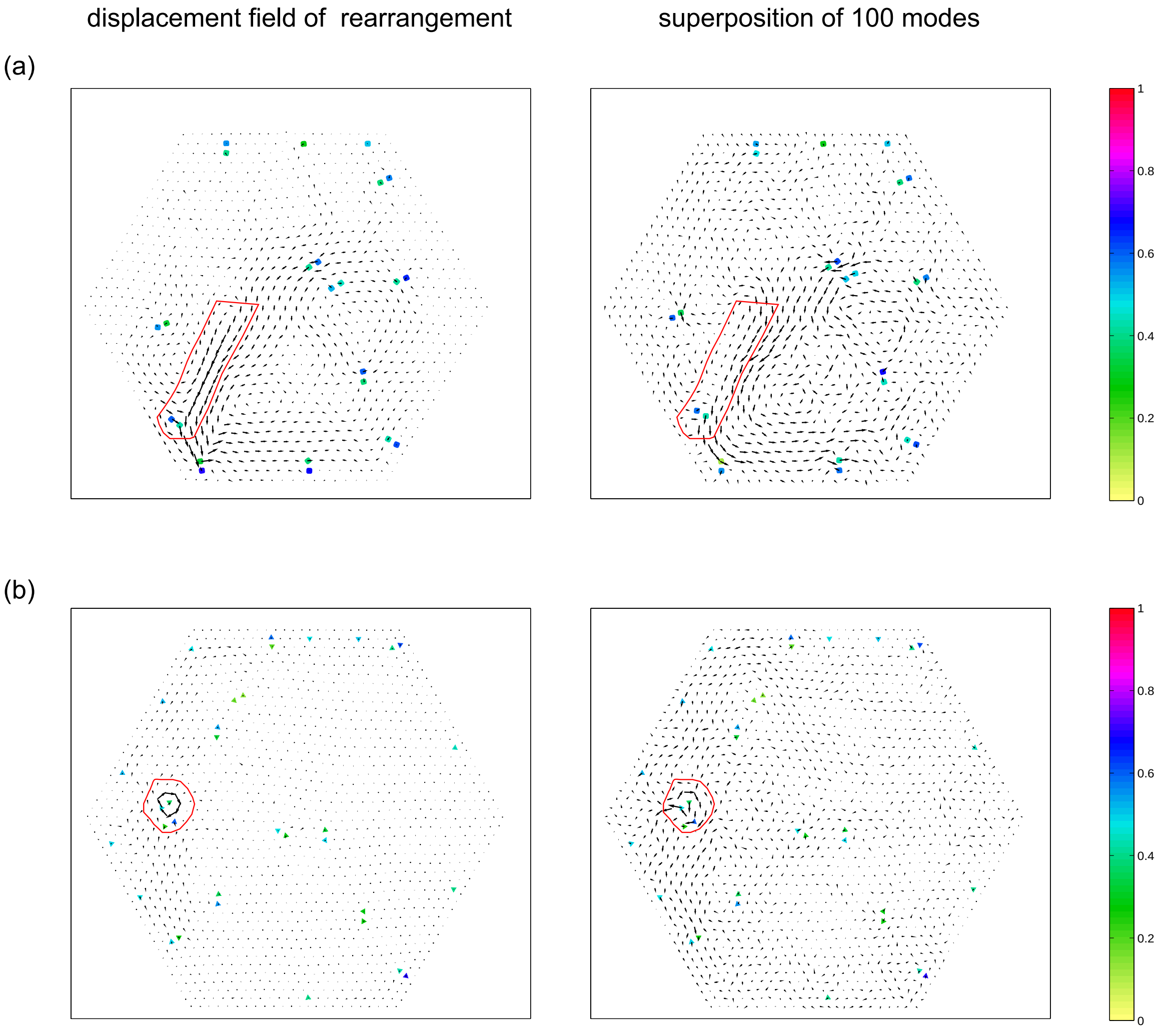

Supplementary Figure 15: Superposition of 100 low-frequency soft modes agrees well with the rearrangement displacement field in (a) a typical square system, and in (b) a typical triangle system. Although the 100 soft modes only makes a small fraction around 3.6% of the total number of modes, the superposition agrees with the displacement very well. The Pearson correlation of the rearrangement region encircled by the red curves is 0.759 for the square system and 0.601 for the triangle system.

basis for defect loop relaxations.

To make a direct comparison, we plot the superposition of 100 low-frequency soft modes side by side with the rearrangement displacement field in Supplementary Figure 15, for both square and triangle systems: clearly the two vector fields are very similar. Although the 100 soft modes only makes about 3.6% of the total number of modes, its superposition describes the rearrangement field very well. In the rearrangement region encircled by the red curves,

the Pearson correlation between the displacement vectors and mode vectors is 0.759 for squares and 0.601 for triangles. The good agreement between soft modes and relaxation fields proves that the system relaxation is driven by the soft modes.
