# Supplementary material for: A universal state and its relaxation mechanisms of long-range interacting polygons
Source: Nat Commun. 2019 Apr 15;10:1737. doi: 10.1038/s41467-019-09795-6 (PMC6465257; doi:10.1038/s41467-019-09795-6)
Supplement: Supplementary file 2 — Description of Additional Supplementary Files [file 41467_2019_9795_MOESM2_ESM.pdf]

**Description of Additional Supplementary Files**

**Supplementary Movie 1**: This movie shows the typical kinetic transitions between different stable states under external perturbations. Each frame shows a stable state and between every two frames, there are 50 cycles of perturbations.

**Supplementary Movie 2**: This movie shows the displacement field of dislocation gliding dynamics for a square-particle system at the effective density of $\varphi = 16.8$. Dislocations with 5 or 7 neighbors are labeled by different colors.

**Supplementary Movie 3**: This movie shows the displacement field of defect-loop relaxation for a triangle-particle system at the effective density of $\varphi = 14.2$. Dislocations with 5 or 7 neighbors are specified by different colors.
